# Intricacies of GABA_A_ Receptor Function: The Critical Role of the β3 Subunit in Norm and Pathology

**DOI:** 10.3390/ijms22031457

**Published:** 2021-02-01

**Authors:** Sergey A. Menzikov, Sergey G. Morozov, Aslan A. Kubatiev

**Affiliations:** 1 Department Russian Academy of Science, Institute of General Pathology and Pathophysiology, 8 Baltiyskaya st., 125315 Moscow, Russia; biopharm@list.ru (S.G.M.); aslan.kubatiev@gmail.com (A.A.K.); 2 Russian Medical Academy of Postdoctoral Education, 2/1 Barrykadnaya st., 123995 Moscow, Russia

**Keywords:** GABA_A_ receptors, β3 subunit, cation–chloride co-transporters, Cl^−^, HCO_3_^−^ATPase, chloride homeostasis, neurodegenerative diseases

## Abstract

Neuronal intracellular chloride ([Cl^−^]_i_) is a key determinant in γ-aminobutyric acid type A (GABA)ergic signaling. γ-Aminobutyric acid type A receptors (GABA_A_Rs) mediate both inhibitory and excitatory neurotransmission, as the passive fluxes of Cl^−^ and HCO_3_^−^ via pores can be reversed by changes in the transmembrane concentration gradient of Cl^−^. The cation–chloride co-transporters (CCCs) are the primary systems for maintaining [Cl^−^]_i_ homeostasis. However, despite extensive electrophysiological data obtained in vitro that are supported by a wide range of molecular biological studies on the expression patterns and properties of CCCs, the presence of ontogenetic changes in [Cl^−^]_i_—along with the consequent shift in GABA reversal potential—remain a subject of debate. Recent studies showed that the β3 subunit possesses properties of the P-type ATPase that participates in the ATP-consuming movement of Cl^−^ via the receptor. Moreover, row studies have demonstrated that the β3 subunit is a key player in GABA_A_R performance and in the appearance of serious neurological disorders. In this review, we discuss the properties and driving forces of CCCs and Cl^−^, HCO_3_^−^ATPase in the maintenance of [Cl^−^]_i_ homeostasis after changes in upcoming GABA_A_R function. Moreover, we discuss the contribution of the β3 subunit in the manifestation of epilepsy, autism, and other syndromes.

## 1. Introduction

Intracellular chloride ([Cl^−^]_i_) and bicarbonate ([HCO_3_^−^]_i_) concentrations are pivotal parameters that control neuronal inhibition and excitation; their effect depends on neuronal specialization and the level of development [1,2,3,4]. γ-Aminobutyric acid type A receptors (GABA_A_Rs) are ionotropic receptors that mediate inhibitory or excitatory neurotransmission, as the net flux of Cl^−^ and HCO_3_^−^ via pores can be reversed by modest changes in the transmembrane concentration gradient of Cl^−^ [5,6,7]. Indeed, in mature neurons, a low [Cl^−^]_i_ renders the reversal potential for GABA (E_GABA_) more hyperpolarized than the membrane potential (E_M_) [8]. The interaction of GABA with synaptic or extrasynaptic GABA_A_Rs leads to Cl^−^ influx into the neurons and the hyperpolarization of E_M_ [9,10,11]. Under certain circumstances (for example, massive activation), GABA_A_ergic signaling can be switched from fast hyperpolarization to long-term depolarization of the E_M_ [12,13,14]. Such paroxysmal depolarizing shifts in the E_M_ during seizures induce Cl^−^ accumulation or the efflux of HCO_3_^−^ through GABA_A_R channels [15,16]. However, in immature and primary sensory neurons—in contrast to mature cells—the elevated [Cl^−^]_i_ is more negative than Cl^−^ equilibrium (E_Cl_^−^), which renders E_GABA_ more depolarized than the E_M_ [17,18]. Here, GABA_A_R activation leads to Cl^−^ efflux from the neurons, depolarizing the E_M_ [19,20]. Thus, E_GABA_ is largely determined by the electrochemical gradient of Cl^−^. Nevertheless, [Cl^−^]_i_ homeostasis is determined by various Cl^−^ conductive systems that may become activated in response to membrane-potential changes (ClC-channels), intracellular Ca^2+^-activated channels (such as anoctamin channels), pH-sensitive Cl^−^ channels (SLC4 and SLC26), and second-active cation–chloride co-transporters (CCCs) [1,6]. However, although Cl^−^ conductive channels may assist in maintaining [Cl^−^]_i_, they cannot generate an electrochemical ionic gradient [21]; electroneutral CCCs play this role first. According to widely accepted continuity, in mature neurons, the K^+^/Cl^−^ cotransporters (KCCs) and (initially) the KCC2 pump Cl^−^ out of cells, which is required for the generation of a rapid GABA_A_R-mediated hyperpolarization of the E_M_ [22,23]. The Na^+^/K^+^/Cl^−^ cotransporter (NKCC1) accumulates Cl^−^ in immature and primary sensory neurons, thereby promoting slow, depolarizing GABA_A_R responses [19]. Generally, there is a dramatic transient shift in GABAergic signaling during development that is associated with the expression levels of CCCs—NKCC1 is downregulated while KCC2 is upregulated [24]. However, while the role of these systems in maintaining ionic homeostasis has been established, their dominant role in the rapid recovery of [Cl^−^]_i_ after changes in upcoming GABA activity remains in doubt, as recent studies have demonstrated [5,25].

Functional GABA_A_Rs are heteropentameric, consisting of five individual subunits encoded by 19 genes that have been characterized and grouped according to their amino acid similarity and named α1-6, β1-3, γ1-3, δ, ε, θ, π, and p1-3 [26,27]. These GABA_A_R subunits are assembled to have a high level of heterogeneity with the general stoichiometry of the 2α, 2β, and 1γ subunits [28]. In several less-abundant subtypes, the γ subunit can be replaced by a δ, ε, or π subunit; the β subunit can be replaced by a θ subunit. The majority of GABA_A_Rs expressed in the human central nervous system (CNS) are α1β2γ2; however, the α3β3γ2 and α2β3γ2 receptor isoforms are also common [29]. Growing evidence suggests that the native assembly of a functional GABA_A_R requires the inclusion of the β subunit [28,30,31]. Furthermore, data from the literature identify the unique role of the β3 subunit in GABA_A_R-mediated inhibition and demonstrate the set of functional properties that distinguish β3 from other β subunits [31,32]. Progress in understanding the role of the β3 subunit in GABA_A_R function has been facilitated by approaches from biochemistry, molecular biology, electrophysiology, and optic genetics [30,31,32,33,34]. Recently, it was reported that the β3 subunit determines the P-type Cl^−^, HCO_3_^−^ATPase (EC 3.6.3.11, Cl^−^-transporting ATPase) [35] that uses the energy of ATP hydrolysis for vanadate-sensitive Cl^−^ transport against an electrochemical gradient [36,37]. The GABA_A_Rs have been identified as key players in processes such as sleep, anxiety, and anesthesia. They may contribute to major disorders of the CNS, including epilepsy, autism, Parkinson’s disease (PD), and Alzheimer’s disease (AD) [38,39,40]. In this review, we discuss achievements in elucidating the role of CCCs and the GABA_A_R β3 subunit in GABAergic neurotransmission. Furthermore, we consider the exceptional role of the β3 subunit in the manifestation of some neurological disorders.

## 2. CCCs and GABA_A_R Activity

### 2.1. Role of KCC2

The maintenance of low [Cl^−^]_i_ is typically attributed to KCC2, which appears to be the primary Cl^−^ extruder in mature neurons [23]. Of the nine KCCs that exist in various cell types, KCC2 is the only one that is expressed in the CNS [41,42,43]. KCC2 is encoded by the solute carrier family 12 member 5 (*SLC12A5*) gene. There are two major isoforms of KCC2: in immature mouse neurons, KCC2a and KCC2b are present in low levels, whereas KCC2b is the major isoform in the adult brain [44]. KCC2 pumps Cl^−^ against its gradient by utilizing the energy electrochemical K^+^ gradient, whose maintenance involves Na^+^, K^+^ ATPase performance [43,44,45,46,47]. KCC2′s net transport activity depends on the chemical gradients of K^+^ and Cl^−^; it is inhibited by the diuretic furosemide (1–2 mM) [48,49,50]. Furosemide produces a greater increase in [Cl^−^]_i_ in adults versus P10 cells, demonstrating that KCC2 activity increases with age [51]. KCC2 has been extensively studied in the context of its influence on GABA_A_R-mediated inhibition because wherever Cl^−^ influx occurs, the transmembrane Cl^−^ gradient is depleted [52]. In particular, the expression of neuron-specific KCC2 is required for the generation of the inwardly directed Cl^−^ electrochemical gradient in CA1 pyramidal neurons of the rat hippocampus [53], as well as other types of neurons [54,55,56]. The hypothesis that KCC2 is necessary for fast Cl^−^-dependent hyperpolarizing inhibition has been supported by results from Northern blot analyses of hippocampal pyramidal neurons [57]. However, despite extensive electrophysiological evidence and a wide range of molecular biological studies on the expression patterns and properties of KCC2 [43,58], ontogenetic changes in [Cl^−^]_i_ and the consequent shift in E_GABA_ remain a subject of debate [59,60].

Indeed, accumulating data on the kinetic properties of KCC2 have called into question their dominant role in the fast recovery of [Cl^−^]_i_ since the time constant is several minutes or longer [48,56,61], while the recovery of GABA_A_R conductance after desensitization occurs at a time constant of approximately several seconds [5,23]. For example, the range of experiences in the various neurons has shown that, after furosemide-induced [Cl^−^]_i_ changes, the KCC2-mediated extrusion of ~5 mM Cl^−^ requires over 5 min [48,56]. These findings are consistent with observations in rat central neurons, indicating that, in contrast to the recovery of E_GABA_, the recovery of [Cl^−^]_i_ after changes is slow (a time constant of ~30 min) [61]. In particular, in different neurons, the recovery of GABA_A_R-mediated conductance after desensitization occurs with a time constant of no more than 13 s [51,62]. Additionally, in vivo experiments in mouse pyramidal neurons demonstrate that the activity-dependent [Cl^−^]_i_ increase of 12 mM that occurs after an epileptic seizure is recovered within less than 30 s [4,63].

### 2.2. Role of NKCC1

Immature neurons express high levels of NKCC1, which increases [Cl^−^]_i_ by using the energy gradient for Na^+^ and K^+^ produced by the Na^+^, K^+^ ATPase [22,64,65]. Earlier studies have demonstrated the essential role of NKCC1 in the transport of Na^+^, K^+^, and Cl^−^ into the cell in various electroneutral stoichiometries, which is selectively blocked by low micromolar concentrations of bumetanide [66,67,68]. Specifically, some evidence supports the involvement of NKCC1 in maintaining high [Cl^−^]_i_ and restoring it after changes [19,69,70,71]. For example, in immature rat neocortical neurons, Cl^−^ accumulation by NKCC1 made up ~37% of the total Cl^−^ transport [66]. In another study, NKCC1 transcripts were detected in practically all thalamus neurons; however, the authors failed to provide evidence for a major contribution of NKCC1 to neuronal Cl^−^ uptake in these neurons [25]. In addition, in chick embryonic motoneurons, it has been found that Cl^−^ accumulation is only partly mediated by NKCC1 [72]. The authors investigated the possible existence of other major Cl^−^ accumulators or anion exchangers that may be responsible for this high [Cl^−^]_i_. The low efficacy of NKCC1 in Cl^−^ uptake has also been observed in sensory neurons. In particular, the use of specific blockers (loop diuretics) of CCCs has indicated that the contribution of NKCC1 to the maintenance of [Cl^−^]_i_ in these neurons accounts for only 23–36% of total Cl^−^ accumulation [73,74]. Moreover, substantial Cl^−^ accumulation persists even in mice lacking NKCC1, while DIDS (a stilbene derivative), which blocks various Cl^−^/HCO_3_^−^ transporters and exchangers, reduced Cl^−^ uptake significantly. However, as noted by the authors, the common Cl^−^/HCO_3_^−^ exchanger (AE2) is unlike the candidate, since the E_M_ in olfactory sensory neurons was found to have a normal amplitude in AE2 knockout mouse pups [74]. The molecular identity of the DIDS-sensitive Cl^−^/HCO_3_^−^ transporter in these neurons was not established.

NKCC1 has been extensively studied in the context of its influence on GABA_A_ergic synaptic transmission. Specifically, in hippocampal pyramidal neurons, in situ hybridization and immunohistochemical results demonstrate greater NKCC1 expression levels during the first postnatal week than in later ages, a temporal sequence that mirrors the changes in GABA responses [75]. Indeed, current studies demonstrate that, in many developing neurons, NKCC1 can contribute to Cl^−^ accumulation, which generates depolarizing GABAergic currents [66,71,72,76]. By reducing NKCC1-mediated Cl^−^ accumulation, bumetanide shifts E_GABA_ to negative potentials, resulting in more effective inhibition [31,77,78]. Specifically, the pharmacological action by bumetanide led to a negative shift in E_GABA_ in high-[Cl^−^]_i_ neurons, in which NKCC1 mRNA was expressed, while it had no effect on low-[Cl^−^]_i_ cells, in which NKCC1 mRNA expression was not detected [79,80]. However, although the molecular properties and mechanism regulation of NKCC1 are well-studied, few reports address the kinetics of NKCC1-dependent Cl^−^ uptake. In particular, in immature rat neocortical neurons and in human neuroblastoma cells, bumetanide-sensitive NKCC1-mediated Cl^−^ uptake has been observed in the time interval of 5–15 min [66,68]. In addition, Gonzalez-Islas et al. showed in chick embryonic motoneurons that Cl^−^ recovery after depletion showed both NKCC1-independent (1–10 min) and NKCC1-dependent (10–50 min) phases [72]. However, the restoration of [Cl^−^]_i_ and GABA_A_R-mediated Cl^−^ conductance after changes had occurred was observed over a period of 3–10 s [71].

### 2.3. CCCs and Neurological Disorders

GABAergic neurotransmission is exceptional in that the polarity of its actions very much depends upon [Cl^−^]_i_ that is highly hesitant, leading to depolarizing and even excitatory actions under some conditions [17,24]. As noted above, most mature neurons in the CNS actively extrude Cl^−^ and thus support a low [Cl^−^]_i_ [1,3], while a return to an immature state in terms of [Cl^−^]_i_ occurs after seizures, spinal cord and brain injuries, massive stimulation, and other pathological conditions [39]. Over the past two decades, the mechanisms underlying the higher [Cl^−^]_i_ accumulation in immature and pathological neurons were explained by the varied efficacy of CCCs [18,45]. Indeed, based on their developmental, cellular, and subcellular patterns of functional expression, CCCs have turned out to be highly diversified factors in ensuring GABA_A_ergic signaling [23]. Disrupting the expression or regulation of NKCC1 or KCC2 during development can change the normal excitation/inhibition balance (E/I), which is critical for proper neuronal circuit development and function [22,23]. Interestingly, even while bumetanide and furosemide were unable to counteract the initial changes in [Cl^−^]_i_, they entirely prevented the secondary rise in [Cl^−^]_i_ during reoxygenation in hippocampal slices from adult mice [69], while the [Cl^−^]_i_ recovery by NKCC1 was observed over 45 min after in vitro ischemia. Kilb et al. demonstrated that bumetanide did not attenuate low-Mg^2+^-induced epileptiform activity, although it did enhance kainate-induced ictal-like events over 60 min [81]. Another study found that bumetanide has no effect on low-seizure activity in neonatal neurons (P5); however, it plays a significant role in progressive Cl^−^ accumulation induced by recurrent seizures [78]. In addition, a residual increase in the GABA_A_R-mediated current in the presence of bumetanide indicates that a persistent elevation of [Cl^−^]_i_ in epileptic neurons is only partially mediated by NKCC1 [72].

The contribution of KCC2 dysfunction and subsequent increases in [Cl^−^]_i_ is also considered as one of the major factors in several neurological disorders. In particular, a downregulation of KCC2 associated with epileptiform activity or after an injury has been observed in several in vivo and in vitro studies [57,62,63]. Rivera et al. found that sustained interictal-like activity in hippocampal slices downregulates the mRNA and protein expression of KCC2 in CA1 pyramidal neurons, which leads to a reduced capacity for neuronal Cl^−^ extrusion [57]. Moreover, it has been shown that even a robust downregulation of KCC2 activity does not abolish inhibitory postsynaptic potentials (IPSPs) if cellular Cl^−^ regulation is challenged by a Cl^−^ load [58] and, besides, that this may take place at a low rate. Finally, Ferrini et al. recently uncovered a gradient in Cl^−^ extrusion capacity via the superficial dorsal horn of the spinal cord (laminae I-II), which remains concealed under a low Cl^−^ load [11]. Under a high Cl^−^ load or a heightened synaptic drive, low Cl^−^ extrusion occurred via the expression of KCC2. However, it is important to note that altering [Cl^−^]_i_ in a time scale of seconds via slowly desensitizing or non-desensitizing GABA_A_Rs may cause the collapse of the Cl^−^ gradient and contribute to the induction or maintenance of pathological conditions (for example, epilepsy) [51,63,64].

In conclusion, important progress in understanding the molecular mechanisms maintaining chloride homeostasis has been achieved, including a definition of a contribution of specific CCCs in GABAergic signaling. Moreover, these studies reveal the dual nature of GABA action, and [Cl^−^]_i_ homeostasis determines the ambivalent behavior of GABA_A_R activity. However, its short-term switching and the rapid recovery of GABA_A_R-mediated Cl^−^ conductance after [Cl^−^]_i_ changes are difficult to explain simply through the driving force of secondary active cotransporters. The expression of CCCs may be necessary for more delayed and long-term processes for maintaining neuronal [Cl^−^]_i_ homeostasis, although CCCs are not the decisive factor in short-term and minor changes. In this regard, the existence of an alternative Cl^−^ transport system is necessary to explain the rapid maintenance of Cl^−^ homeostasis.

## 3. Role of GABA_A_R/Cl^−^, HCO_3_^−^ ATPase

Historically, it has been believed that, unlike the plasmalemmal ATPases, which utilize the potential energy of ATP hydrolysis for the active transport of ions against their electrochemical gradients [82], the GABA_A_Rs are passively permeable to anions [83,84]. However, early research suggested that GABA_A_R function can involve both ATP-binding [85,86] and ATP-hydrolysis processes [87]. Specifically, electrophysiological studies have identified the presence of atypical GABA_A_Rs that participate in Cl^−^-transport against an electrochemical gradient in mammalian vestibular Deiters’ neurons [88,89]. It has been suggested by the authors that these “receptors” are GABA-activated chloride extrusion pumps, where the energy for chloride extrusion is provided by ATP in a phosphorylation step within the extrusion cycle. A recent study has directly shown that GABA_A_R can operate as a P-type ATPase that transports Cl^−^ ions by consuming ATP energy [35,90,91]. Indeed, it was found that β3 (unlike the α and γ subunits) is catalytic, and determines the hydrolysis of ATP [35]. Activation of the ATPase requires the presence of 5 mM Cl^−^ and 25 mM HCO_3_^−^ (Cl^−^, HCO_3_^−^ATPase) in the experience medium (20 mM Hepes–Tris pH 7.3, 2 mM Mg^2+^-ATP), although other anions can also stimulate the enzyme in the following range: Cl^−^ ≥ Br^−^ > I^−^ ≥ F^−^ [92,93]. Nonetheless, the presence of a physiological concentration of HCO_3_^−^ is an absolutely necessary condition for both the stabilization of ATPase activity and ATP-dependent Cl^−^ transport via membranes; Na^+^ and K^+^ have no effect. ATP in the presence of Mg^2+^ is the most effective hydrolyzed nucleotide (ATP > UTP > CTP > ADP). Other divalent cations can replace Mg^2+^ in the following order: Mg^2+^ > Mn^2+^ > Co^2+^ > Al^2+^ > Cd^2+^ [92,93]. The β3-containing GABA_A_R ensembles can catalyze the auto-phosphorylation of a high-energy aspartate residue during ATP hydrolysis; this is inhibited by micromolar concentrations of γ-phosphate analogs such as *o*-vanadate or metal fluoride complexes [35,90]. The enzyme is sensitive to low concentrations of stilbene-derivative compounds (SITS and DIDS) and to the loop diuretic furosemide; it also has low sensitivity to another diuretic, bumetanide [91,94]. An important property of this enzyme is the regulation of its catalytic properties by GABA_A_ergic ligands (benzodiazepines and barbiturates) and its inhibition by specific GABA_A_R blockers—bicuculline or picrotoxin [35,92,93]. Furthermore, the GABA_A_R-coupled Cl^−^, HCO_3_^−^ ATPase and its Cl^−^-transporting performance are highly sensitive to phenol and phenol derivatives, which distinguishes it from other transport ATPases [90].

GABA_A_R is permeable to two physiological anions: Cl^−^ and HCO_3_^−^ [4,7,13,14,15]. Under physiological conditions, Cl^−^ is the primary charge carrier across the receptor pore. However, under such conditions (as example, massive activation), HCO_3_^−^ ions that flow in the opposite direction (at an HCO_3_^−^/Cl^−^ ratio of 0.2/0.3) [83,84] can contribute to the net current via GABA_A_R, depending on both pH and [Cl^−^]_i_ [4,5]. The Cl^−^, HCO_3_^−^ ATPase functions with anions at a ratio of ~5:1 (HCO_3_^−^ to Cl^−^), indicating a difference in the pumping processes and passive channel conductance, while this ATPase displays a diverse number of properties in its dependence on specificity neurons. For instance, HCO_3_^−^ ions do not play a key role in the activation of the Cl^−^ATPase in the primary sensory neurons, but solely in its stabilization, unlike mature neurons, where they play an important role in GABA_A_R-mediated depolarization and in Cl^−^, HCO_3_^−^ ATPase activation [87]. Thus, the role of the ATPase in bicarbonate transport and in membrane hyperpolarization must be clarified in future research. Furthermore, it seems necessary to establish the properties and roles of the ATPase in immature neurons. In addition, the β3 subunit, reconstituted into proteoliposomes, participates in ATP-dependent Cl^−^-transport over a short period (5–30 s) and then reaches a plateau [35]. To investigate this, we observed the inhibition of ion-pumping processes by γ-phosphate analogs. Thus, dependent on changes in [Cl^−^]_i_, the enzyme begins to pump anions into and out of the cell during this time period to reestablish anionic electrochemical gradients for upcoming GABA activity; therefore, this activity may have important physiological significance. Recent research confirms that this ATPase plays an important role in maintaining [Cl^−^]_i_ levels and shows that enzymatic activity is elevated during seizures [90] and returns to baseline levels when seizures cease. In addition, there is some evidence to support the theory that rapid activity-induced elevation of [Cl^−^]_i_, via the GABA_A_ receptor, contributes to certain disorders (for example, epileptic activity) [4,16].

**Figure 1:**
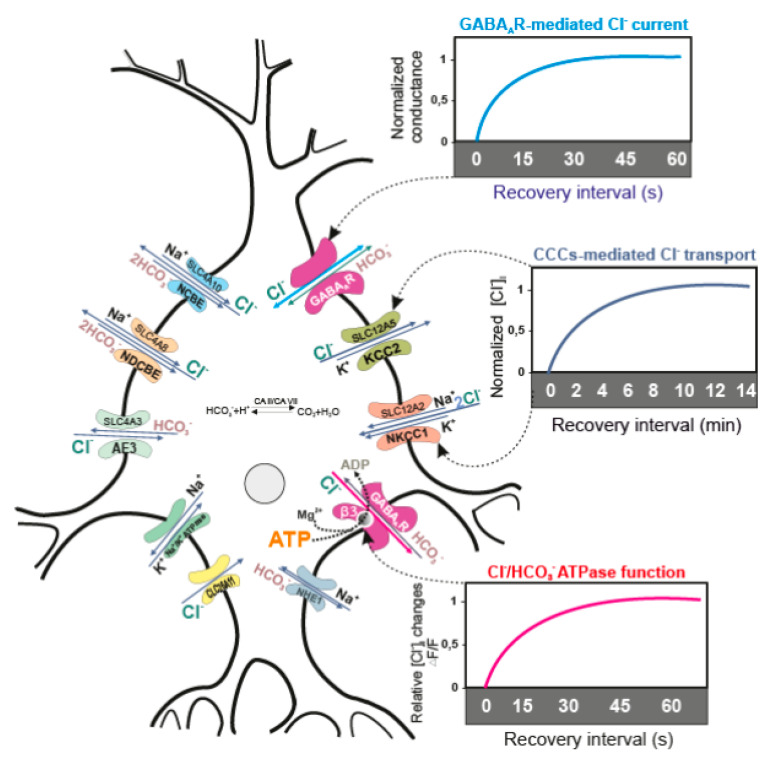
Performance of transporters and recovery of the receptor function. Neuronal [Cl^−^]_i_ is maintained mainly by neutral active CCCs (KCC2 and NKCC1), and less by Cl^−^ channels (*SLC26* family) and exchangers (*SLC4* family—AE3, NCBE or NDCBE). CCCs move the Cl^−^ ions by utilizing the energy electrochemical Na^+^ and K^+^ gradient, whose maintenance involves Na^+^, K^+^ATPase. CCCs need at least 5 min to recover [Cl^−^]_i_ [48,57,68,69,70,71], while the GABA_A_R-mediated conductance may recover during approximately 10–15 s after [Cl^−^]_i_ changes [51,61]. After purification and reconstitution into proteoliposomes, the β3-containing GABA_A_R homopentamer participates in ATP-consuming Cl^−^ transport for a short period (~30 s) and then reaches a plateau [35]. The bottom panel shows the changes of [Cl^−^]_i_ after application of Mg^2+^-ATP in the experimental medium containing proteoliposomes with embedded GABA_A_R/Cl^−^, HCO_3_^−^ATPase, as well as the fluorescent dye for chloride.

Thus, β3-containing GABA_A_Rs possess bifunctional properties—that is, they can operate in two different modes: as a GABA_A_-gated Cl^−^-channel or as an anion-transporting P-type ATPase. This indicates that this enzyme not only maintains chloride homeostasis in various neurons, but can also facilitate a change in GABAergic neurotransmission from excitation to inhibition, which is crucial in the CNS (Figure 1). However, it should be noted that the kinetic properties of Cl^−^, HCO_3_^−^ ATPase have only been observed in vitro, which may not fully reflect in vivo studies. Therefore, until conclusive in vivo evidence is presented to confirm this phenomenon, its importance and relevance will remain in question.

## 4. Role of the β3 Subunit in GABA_A_R Function

All the GABA_A_Rs without a β subunit are inactive, but many studies have noted the crucial importance of the β3 subunit in the functional properties of GABA_A_Rs. These properties distinguish β3 from both the β1/β2 subunits and the α and γ subunits [31,95]. In particular, the chimeric β3—in contrast to α2 and γ2 subunits—determines the ionic selectivity of recombinant GABA_A_R [32]. Moreover, the β3 and (to a lesser extent) β1 subunits can form functional homomeric ion channels in the various cells that are not only modulated by GABA, but also inhibited by picrotoxin and activated by pentobarbital [96,97]. While all five subunits are involved in the overall design of the pore, only the two β3 subunits are responsible for pore formation; this implies the importance of features that are absent in the β3 M1–M2 loop or the M2 domain, but present in both the α2 and γ2 counterparts [98]. Through a chimeric approach, four amino acids (glycine 171, lysine 173, glutamate 179, and arginine 180) in the N-terminal domain of the β3 subunit have been identified as mediating the functional cell-surface expression of this subunit, unlike β2, which is retained within the endoplasmic reticulum. In addition, immunofluorescence studies focusing on the β3 subunit have shown that (in contrast to homomeric α1, β2, or γ2L subunits) this protein can access the cell surface via homomeric expression [99].

Recently, it was found that the crystallized human GABA_A_R β3 homopentamer channel forms a closed gate at the base of the pore, representative of a desensitized state [100]. Furthermore, the β3 subunit is the molecular target for insecticides and phenol derivatives; other subunits (α1, α6, or γ2) differentially modulate binding to counter compound-dependent specificity and selective influence [90,101]. Moreover, a β3 subunit in the contrast to other β subunits is required alone or with another subunit (α1 or γ2) for the assembly of the [^3^H]ethynylbicycloorthobenzoate binding site. The β subunits are the primary substrates for various kinases in neurons. However, the regulation of GABA_A_R β subunits—as distinct from that of other subunits—may vary depending on the type of kinase [97]. Specifically, β3 is basally phosphorylated at the S408/S409 amino acid residue and is distinct from the β2 subunit in that it is phosphorylated by calcium/calmodulin-dependent kinase [102,103].

**Figure 2:**
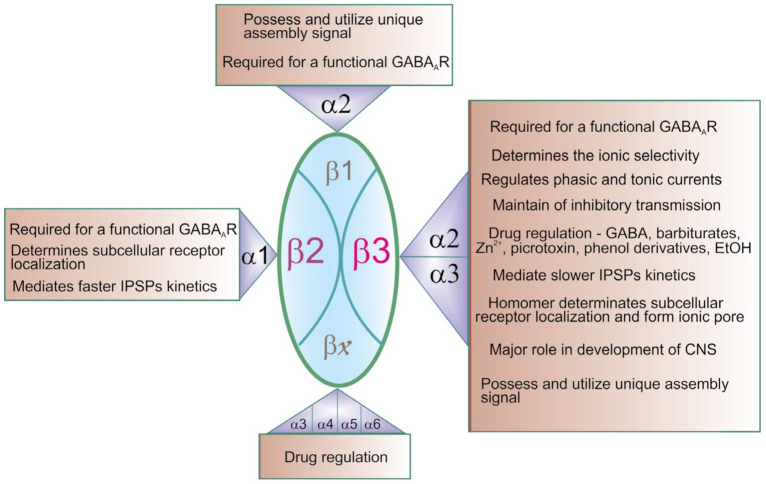
Contribution of the β subunits in heterogeneity and functional properties of γ-aminobutyric acid type A receptors (GABA_A_Rs). In the central nervous system (CNS), the majority of expressed GABA_A_Rs are β2-containing (α1β2γ2) and β3-containing (α2β3γ2 and α3β3γ2) ensembles [27,28]. The β1-containing subtypes are in fewer in number. The β2-containing subtypes demonstrated a low range of properties. The β3-containing ensembles appear to have diverse properties (including maintaining properties of the inhibitory transmission and the determination of the ionic selectivity). The βx-containing ensembles are fewer in number and insufficiently defined.

Using both CRISPR/Cas9 and optogenetic approaches, it was found that the presence of the β subunits (β1, β2, and β3) is absolutely necessary for the native assembly of a functional GABA_A_R [31]. While the knockout of both β1 and β2 did not change the inhibitory synaptic transmission, the presence of β3 alone was sufficient to maintain proper inhibitory transmission in the hippocampus. Indeed, when β3 is knocked out, either alone or in combination with another β isoform, inhibitory currents are depressed. The role of β3 was observed to be even more critical in the absence of β1 and β2 or all endogenous β subunits where expression of β3 alone is sufficient to maintain or restore inhibitory currents, respectively. In addition, the expression of β3 in the β1–β2 subunit knockout can fully restore responses to control levels [31]. This confirms that, out of the three β subunits, β3 is the most important for proper inhibitory transmission. It is possible that this is precisely due to the presence of Cl^−^, HCO_3_^−^ATPase in the structure of the β3 subunit and that it is able to maintain the GABA-mediated inhibitory currents. Further studies should show what other β subunits have the Cl^−^, HCO_3_^−^ATPase activity and affirm the uniqueness of the β3 subunit. The uniqueness of the β3 subunit also indicates its kinetic properties. In particular, results show faster kinetics in the β3 knockout GABA_A_Rs and confirm that the β3 subunit preferentially associates with α2 and α3 subunits to mediate slower IPSP decay kinetics and, therefore, longer-lasting inhibition. Indeed, it has been shown that the β3 subunit preferentially associates with the α2/α3 subunits; this is distinct from β2, which couples mainly with α1 [26,27,28,29] (Figure 2).

Moreover, the decay kinetics of GABA_A_R depend on the identity of the particular α subunit isoform expressed, with α1-containing receptors having faster kinetics than α2/α3-containing receptors [30,104,105], while both the β3 subunit and α subunit determine the GABA potency [106,107]. In addition, the essential role of the β3 subunit in mediating spiny projection neuron tonic currents has been demonstrated using conditional β3 subunit knockout (β3f/f^Drd2^) mice [34]. The GABA_A_R β3 subunit gene (*GABRB3*) is known to play a major role in the development of the CNS, being the major β isoform in multiple regions in the prenatal and neonatal brain [106]. A developmental deficit of GABA_A_R function affects neurogenesis and maturation of the neuronal network [108].

## 5. The β3 Subunit and Human Diseases

### 5.1. Epilepsy

Epilepsy is a group of neurological disorders characterized by recurrent epileptic seizures with a range of etiologies and comorbidities [108]. A number of studies have demonstrated that *GABRB3* gene mutations are associated with a broad phenotypic spectrum of epilepsies, and that reduced GABA_A_R function, causing GABA_A_ergic disinhibition, represents the relevant disease mechanism [109,110,111,112]. In particular, the Epi 4K consortium has identified four de novo mutations in the *GABRB3* in children with epileptic encephalopathies [113]. Research has found that mutations in GABA_A_R subunit genes (in particular, *GABRB3*) are associated with idiopathic epilepsy, including childhood absence epilepsy, juvenile myoclonic epilepsy, and other syndromes [109,110,111,114]. As an example, the *GABRB3* mutation G32R, which is associated with childhood absence epilepsy, alters the expression of α1β3γ2L GABA_A_R, as well as channel-gating [109]. Janve et al. also demonstrated that the epileptic encephalopathy de novo gene *GABRB3* (D120N, E180G, and Y302C) impairs GABA_A_R function [111]. In addition, the GABA_A_R coupling junction and pore *GABRB3* mutations are linked to early-onset epileptic encephalopathy [115,116]. In the hippocampus of patients with temporal lobe epilepsy, expression of GABA_A_R β subunits (including the β3 subunit) was increased [117]. Interestingly, it was found that hyperglycosylation [118] and reduced GABA currents can alter receptor expression and channel-gating of mutated *GABRB3* polypeptides to reduce childhood absence epilepsy [109].

**Figure 3:**
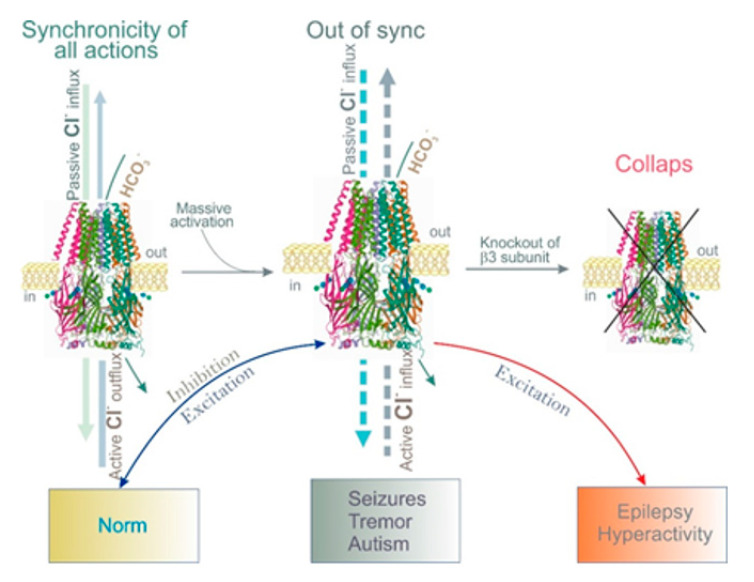
Role of the β3 subunit in dissipated and generated Cl^−^ gradients in norm and pathology. (**Left**) Schematic representation showing synchronicity work of passive and active Cl^−^ fluxes via human GABA_A_B3R homopentamer (Protein Data Bank, 4COF) [93]. (**Middle**) Impaired synchronicity of multidirectional Cl^−^ fluxes across receptor pores in certain circumstances (massive activation by GABA or other ligands). (**Right**) Collapse of Cl^−^ moving via receptor pores lacking the β3 subunit.

The GABA_A_R β3 subunit is widely expressed in immature and adult brains in circuits involved in seizure generation, such as the cortex, hippocampus, and thalamic reticular nucleus [119]. Furthermore, it was found that mutant residues are part of conserved structural domains, such as the Cys-loop (L170R) and the M2-M3 loop (A305V), which form the GABA binding/channel-gating coupling junction and the channel pore (T288N), which are functionally coupled during receptor activation. In addition, mice lacking the β3 subunits exhibit thalamic disinhibition, a major reduction in GABA_A_R expression, and seizures that are associated with learning and memory deficits, poor motor skills on a repetitive task, hyperactivity, and a disturbed rest–activity cycle [120]—all features characteristic of children affected by this neurological disorder [110,111,112,113,114,116]. Null-β3 mice produce fewer functional GABA_A_Rs; pharmacological evidence indicates that other β subunits do not compensate for the absence of β3 [120]. Moreover, in model epilepsy, there is a modulation of the expression of the GABA_A_R β1 and β3 subunits; as a result, Cl^−^ extrusion is impaired, and GABA_A_R-mediated depolarization appears [121,122,123,124]. These data highlight the importance of the β3 subunit in the appearance of epilepsy (Figure 3).

### 5.2. Autism

Autism spectrum disorders (ASDs) are a group of complex disorders of neurodevelopment characterized by repetitive behaviors and difficulties with social interaction and verbal and nonverbal communication [125]. With ASD, deficits in social cognition and related cognitive functions may result from reduced synchronization between brain regions [126]. A possible explanation for ASDs is the disturbance of the delicate balance between excitation and inhibition in the developing brain, which may profoundly impact neurobehavioral phenotypes. An imbalance between excitation and inhibition may result from an increase in glutamatergic signaling (excitatory) or reduced inhibitory GABA_A_ergic signaling [127,128,129]. Evidence connecting GABA_A_Rs in the etiology of ASD was first provided by genetic studies revealing submicroscopic abnormalities in the chromosomal locus 15q11–q13, which contains the *GABRB3*, *GABRA5*, and *GABRG3* genes encoding the GABA_A_R β3, α5, and γ3 subunits, respectively [126,130,131]. In particular, the single-nucleotide polymorphism present in *GABRB3* (rs2081648 and rs1426217) genes demonstrates significant associations between ASD and age- and gender-frequency-matched typically developing controls [127]. Locus 15q 11–13 duplications have been observed in ASD patients; association studies in idiopathic autism patients have found significant evidence for a susceptibility allele in the *GABRB3* gene [131]. In addition, *GABRB3* rs2081648 polymorphisms are associated with symptom-based deficits in social interaction and in sensorimotor and somatosensory coordination, visual response, imitation, activity level, and adaptability [132]. Although a mutation in the *GABRB3* gene was associated with a 3–6 times greater risk of ASD with epilepsy [127], the mutation of other GABA_A_R subunits, including the β1 and α4 subunits, has also been coupled with ASD within various ethnic groups [133]. In addition, knockout mice for *GABRA5* and *GABRG3* have a normal phenotype, while *GABRB3* knockouts have severe neurological abnormalities, hypersensitivity to tactile stimuli, and defects in social and exploratory behavior [120,126]. The expression of GABA_A_R subunits (including the ASD-relevant *GABRB3*) was also altered in the forebrain of young and adult Engrailed*-2* knockout mice [134]. In particular, the GABA_A_Rs are reduced in various brain regions, with *GABRB3* significantly altered in the parietal cortex and cerebellum. Overall, β3-containing GABA_A_Rs might be important players in the etiology of ASD pathology (Figure 3).

### 5.3. Alzheimer’s Disease (AD)

AD is a progressive neurodegenerative disorder that leads to the loss of cognitive functions such as executive function, learning, and memory [135]. AD is associated with a widespread loss of synapse density and continuous degeneration of cholinergic and glutamatergic pathways. Although the disruption of excitatory pathways is broadly accepted, inhibitory GABAergic pathways are generally thought to be well preserved in AD [135], while, with regard to progressive dementia, AD is characterized by an increased incidence of seizure activity. This was originally believed to be a secondary process occurring as a result of neurodegeneration. However, recent research has suggested that alterations in the E/I balance occur in AD and may be a primary mechanism contributing to the cognitive decline seen with AD [136]. Using immunohistochemistry and laser-scanning confocal microscopy, brain region-specific and cell layer-specific alterations were found in the expressions of the β2 and β3 subunits in the human hippocampus, entorhinal cortex, and superior temporal gyrus in AD cases [137,138]. In particular, the expression of three GABA_A_R subtypes was altered—α2 was upregulated, while the α5 and β3 subunits were downregulated [139]. Moreover, lower levels of GABA_A_R β2 mRNA in the prefrontal cortex of the AD brain [138]—along with lower levels of β3 mRNAs in the AD hippocampus [135]—suggest that some GABA_A_R subtypes may have an altered functional profile in AD. This contrasts with the β1 subunit, which was well-preserved.

### 5.4. Parkinson’s Disease (PD)

PD is the most common neurodegenerative movement disorder. Its clinical manifestations include motor symptoms (bradykinesia, rigidity, postural instability, and resting tremors) and a variety of nonmotor symptoms (particularly sleep and behavioral disturbances) [140,141]. It is generally thought that bradykinesia results from the loss of dopaminergic neurons in the substantia nigra pars compacta and subsequent striatal dopamine depletion [142]. However, it has recently been suggested that GABA plays a modulatory role in the pathophysiology of PD that is independent of dopaminergic medication [143,144,145]. In addition, the GABA_A_R/Cl^−^, HCO_3_^−^ATPase from rat brain is involved in the phenol-induced manifestations of both head-twitching and tremors [94]. Moreover, an interaction between dopamine and GABA was established in the basal ganglia [146]. In particular, dopamine in the absence of GABA can directly modulate recombinant GABA_A_Rs via binding with the β3 subunit [147]. In this regard, clarification of the role of the β3 subunit in the appearance of PD may be of particular interest.

## 6. Concluding Remarks

Since the 1970s, GABA_A_Rs have been considered as passively permeable, ligand-regulated Cl^−^ channels that—after binding to GABA—mediate either hyperpolarization or depolarization of transmembrane potential. Among all subunits, an exclusive role in GABA_A_R functioning has been determined only for the β3 subunit. Research data on the properties of GABA_A_Rs highlight that the β3 subunit is an independent structure that can singly form an ion pore; importantly, it is also a key polypeptide that facilitates inhibitory neurotransmission. Moreover, recent findings indicate that β3-containing GABA_A_Rs are P-type ATPases, allowing us to review them as bifunctional systems. Other β subunits may also possess ATPase activity. These properties of GABA_A_Rs necessitate a special look at their role in neurological disorders—for instance, the violation of passive GABA_A_R-mediated Cl^−^ currents or ATP-dependent Cl^−^ pumping changes chloride homeostasis. In summary, advances in understanding how GABA_A_Rs operate have drawn a much more complex picture than what existed previously. New methods (particularly optic genetic methods) and approaches (including the use of genetically modified animals) provide multiple avenues for future research to elucidate the mechanisms of GABA_A_R function and reveal how its dysfunction leads to disease. We believe that the GABA_A_R β3 subunit may serve as a primary pharmacotherapeutic target. We hope that this paper will foster interest in clarifying the posttranslational mechanisms of its regulation.
